# Vivisectionists Scared Up

**Published:** 1896-08

**Authors:** 


**Scared Up.**—The cranks at Washington are beseeching congress to pass an anti-vivisection law. If passed, each State will follow suit by legislative enactment, and all laboratories of research in biology will be closed. At Atlanta, during the meeting of the American Medical Association last month, the following resolution was adopted as a sort of counter-stroke:

Whereas, the members of the American Medical Association recognize the fact that the developments of scientific medicine have resulted largely from experiments upon the lower animals; and

Whereas, anesthetics are habitually administered to animals subjected to painful experiments; and

Whereas, restrictive legislation is in our opinion unnecessary and opposed to the continued progress of medical science; and

Whereas, it is an unjust reflection upon the humanity of those engaged in animal experimentation to enact laws requiring them to use anesthetics and appointing inspectors to see that they do so; and

WHEREAS, far more unnecessary pain is constantly being inflicted upon the lower animals for sport and game than in biologic and pathologic laboratories; and

WHEREAS, no evidence has been presented by those who advocate restrictive legislation showing that abuses exist in the District of Columbia; and

WHEREAS, results of great practical importance have been obtained by experiments on the lower animals made in the government laboratories in the District of Columbia; therefore be it

*Resolved*, That the American Medical Association earnestly protests against the passage of Senate Bill No. 1552, entitled, "A Bill for the further prevention of cruelty to animals in the District of Columbia," or any modification of this bill, unless it shall first be shown by an impartial investigation that cruel and unnecessary experiments are being performed in the District of Columbia, and that existing laws do not provide suitable punishment for the cruelty to the domestic animals.

*Resolved*, That copies of these resolutions, attested by the signatures of the president of the American Medical Association, and of its committee appointed to draft these resolutions, be sent to the chairman of the Committees on the District of Columbia, in the house of representatives and senate of the United States.

(Signed) DR. NICHOLAS SENN,
DR. WM. OSLER,
DR. GEO. M. GOULD,
DR. J. MCFADEN GASTON,
DR. DONALD MACLEAN.

